# Mosquito blood-feeding patterns and nesting behavior of American crows, an amplifying host of West Nile virus

**DOI:** 10.1186/s13071-021-04827-x

**Published:** 2021-06-22

**Authors:** Sarah S. Wheeler, Conor C. Taff, William K. Reisen, Andrea K. Townsend

**Affiliations:** 1 Sacramento-Yolo Mosquito and Vector Control District, 8631 Bond Road, Elk Grove, CA 95624 USA; 2 Cornell Lab of Ornithology and Department of Ecology and Evolutionary Biology, Cornell University, Ithaca, NY 14850 USA; 3 Department of Pathology, Microbiology and Immunology, School of Veterinary Medicine, University of California, Davis, CA 95616 USA; 4 Department of Biology, Hamilton College, 198 College Hill Rd, Clinton, NY 13323 USA

**Keywords:** American crow, *Corvus brachyrhynchos*, *Culex pipiens*, *Culex tarsalis*, West Nile virus, Blood meal identification, Molecular sexing, Microsatellite analysis

## Abstract

**Background:**

Although American crows are a key indicator species for West Nile virus (WNV) and mount among the highest viremias reported for any host, the importance of crows in the WNV transmission cycle has been called into question because of their consistent underrepresentation in studies of *Culex* blood meal sources. Here, we test the hypothesis that this apparent underrepresentation could be due, in part, to underrepresentation of crow nesting habitat from mosquito sampling designs. Specifically, we examine how the likelihood of a crow blood meal changes with distance to and timing of active crow nests in a Davis, California, population.

**Methods:**

Sixty artificial mosquito resting sites were deployed from May to September 2014 in varying proximity to known crow nesting sites, and *Culex* blood meal hosts were identified by DNA barcoding. Genotypes from crow blood meals and local crows (72 nestlings from 30 broods and 389 local breeders and helpers) were used to match mosquito blood meals to specific local crows.

**Results:**

Among the 297 identified *Culex* blood meals, 20 (6.7%) were attributable to crows. The mean percentage of blood meals of crow origin was 19% in the nesting period (1 May–18 June 2014), but 0% in the weeks after fledging (19 June–1 September 2014), and the likelihood of a crow blood meal increased with proximity to an active nest: the odds that crows hosted a *Culex* blood meal were 38.07 times greater within 10 m of an active nest than > 10 m from an active nest. Nine of ten crow blood meals that could be matched to a genotype of a specific crow belonged to either nestlings in these nests or their mothers. Six of the seven genotypes that could not be attributed to sampled birds belonged to females, a sex bias likely due to mosquitoes targeting incubating or brooding females.

**Conclusion:**

Data herein indicate that breeding crows serve as hosts for *Culex* in the initial stages of the WNV spring enzootic cycle. Given their high viremia, infected crows could thereby contribute to the re-initiation and early amplification of the virus, increasing its availability as mosquitoes shift to other moderately competent later-breeding avian hosts.
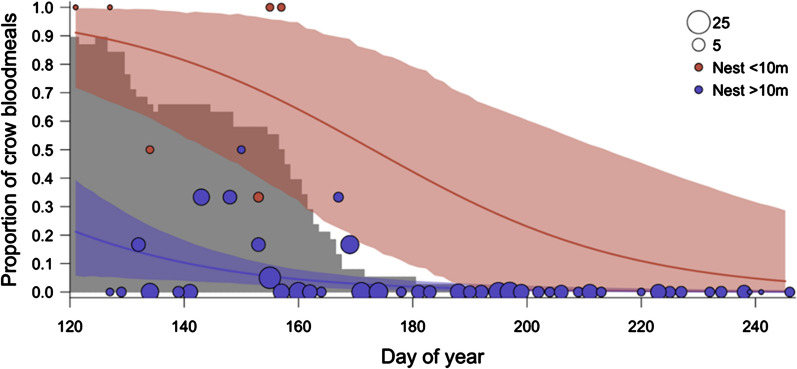

**Supplementary Information:**

The online version contains supplementary material available at 10.1186/s13071-021-04827-x.

## Background

American crows (*Corvus brachyrhynchos*; “crows” hereafter) are a key indicator species for the invasion, spread, and seasonal amplification of West Nile virus (WNV; Flaviviridae: Flavivirus; [1]). Extensive crow die-offs have been associated with WNV epidemics in North American [2, 3], and WNV-positive crows frequently predominate in avian mortality surveillance programs [4, 5]. Crows are the most susceptible of the North American corvids to WNV, producing some of the highest viremias among passeriform birds and exhibiting high mortality after infection (~ 100% in experimental infection trials [6, 7]). Crows reach their highest densities in human-dominated landscapes [8], and declines in crow populations across large geographic scales correlate with the intensity of human WNV epidemics [9]. In addition, crow carcass clusters generally delineate human case clusters [5, 10], and WNV-positive mosquitoes are nearly 20 times more likely to be present at residences where crow carcasses are detected [11]. Considered in concert, these studies indicate that crows are a key amplifying host for WNV in North America.

Despite these lines of evidence, the importance of crows in the WNV cycle has been called into question because of their consistent underrepresentation in studies of *Culex* blood meal sources [12–14]. Numerous blood meal identification studies of *Culex* vectors across the US have reported that crow blood meals are rare or absent (e.g. Alabama [15], Texas [16], New Jersey [17], New York [18], and California [19, 20]), although > 20% of *Cx. pipiens* complex blood meals collected from Sutter County, California, were of crow origin [21, 22]). In contrast, several less-competent species, including the American robin (*Turdus migratorius*), house finch (*Haemorhous mexicanus*), and mourning dove (*Zenaida macroura*) [17, 19, 20, 23–25], were overrepresented as blood meal sources, leading to the hypothesis that these species have a greater impact on WNV epidemiology than crows [12]. The apparent underutilization of crows in *Culex* blood meals is enigmatic, because crows in many of these populations clearly have acquired and continue to acquire WNV infections [4, 26–28]. Some authors have proposed alternative mechanisms for infection in crows, such as bird-to-bird transmission within roosts [17] or scavenging on infected carcasses [16]. However, bird-to-bird transmission was insufficient to amplify (or even maintain) WNV infection in a 7000-bird communal crow roost [27], and the occasional infected, scavenged carcass seemed an unlikely route for the widespread infection indicated by the high prevalence of crows in dead bird surveillance programs [1, 4, 29]. It seems more likely, therefore, that crows acquire the majority of their infections through bites of infectious mosquito vectors, despite their underrepresentation in blood meal studies.

Host utilization by questing mosquitoes can reflect both a preference for specific host species and the extent to which they encounter that host [30–32]. Crows do not appear to be avoided by host-seeking *Culex*: for example, an experiment that controlled for encounter opportunity indicated that American crows were preferred by one *Culex* species (*Cx. erythrothorax*) over other avian hosts (American robins, mourning doves, and house finches) and were utilized proportional to their availability by other *Culex* species (*Cx. tarsalis* and *Cx. quinquefasciatus* [30]). Encounter opportunity—as might arise through nesting behavior—might be a more important determinant of crow-vector interactions than avoidance or preference.

Nesting is likely to increase blood-feeding opportunities for host-seeking mosquitoes if nestlings and breeding adults are readily available hosts [23, 33–35]. Adult birds that are incubating eggs or brooding nestlings (in crows, the female breeders) may be unable to evade host-seeking mosquitoes; nestlings (when not sheltered by their parents) could be even more vulnerable to vectors because of their immobility and incomplete plumage [36]. Indeed, mosquito host shifts among avian species have been attributed to the timing of nesting [22, 37], and seasonal shifts from birds to mammals have been attributed to the termination of the avian breeding season [25, 33, 35, 38]. Breeding crows could be particularly attractive as early season *Culex* hosts for two reasons. First, they are large-bodied birds that breed in family groups comprising a breeding pair, adult helpers, and nestlings [39–41]; in aggregate, these family groups might be particularly attractive to mosquitoes because of the carbon dioxide and heat that they emit [34]. Second, crows often nest in the canopy of the tallest available trees, and *Culex* host-seeking increases with canopy height [42]. If *Culex* do feed upon nesting crows, they could play an important role in the early season amplification of WNV, as crows produce high-titered viremias [6, 7] and are highly competent amplifying hosts [43] for WNV.

The current study was designed to clarify the role of crows as blood meal hosts for *Culex* mosquitoes before, during, and after the crow breeding period. First, we identified the host source of blood meals from mosquitoes using genetic analyses and then calculated the proportion of blood meals that originated from crow hosts in a study population in Davis, California, from May to September 2014. We predicted that the proportion of crow blood meals would be highest from early May to mid-June, when incubating crow female breeders and nestlings are particularly vulnerable to host-seeking mosquitoes. Conversely, we predicted that detections of crow blood meals would decrease in late June to September, when crow fledglings and adults are more mobile. We further examined the importance of nesting crows as hosts by assessing the prevalence of crow blood meals in resting *Culex* females as a function of distance at capture to active crow nests, predicting that the likelihood of crow blood meals would increase with proximity to an active nest. Finally, we used microsatellite analysis to match DNA from mosquito blood meals to specific crows sampled in the focal population to determine the relative contributions of nestlings, fledglings, adult helpers, and adult breeders as mosquito hosts.

## Methods

### Field site and crow sampling

**Fig. 1:**
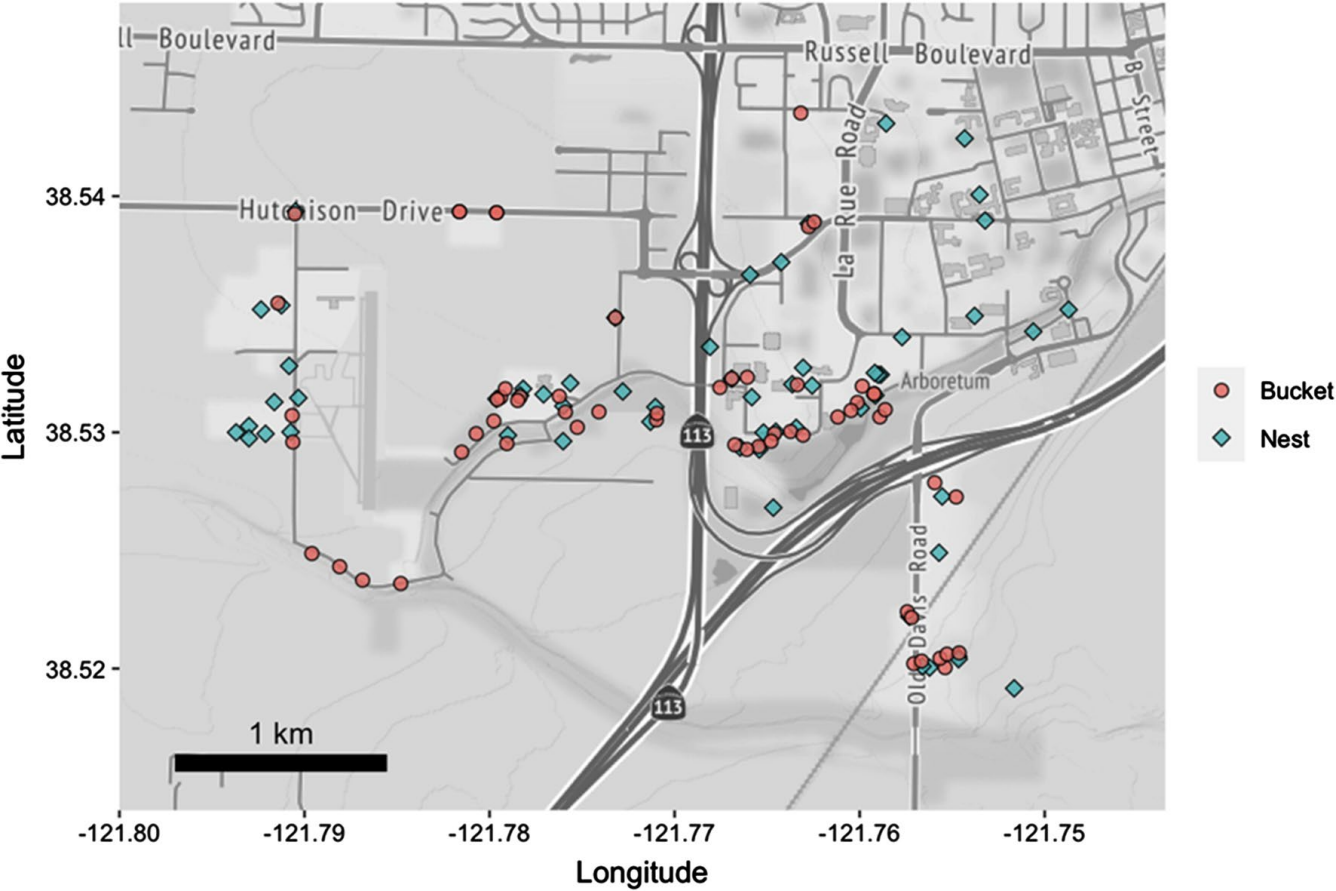
Map of study site in Davis, California. Sampled crow nests indicated by blue diamonds. Red circles indicate locations of the artificial resting sites for mosquito collection (“buckets”). Map tiles by Stamen Design, under CC BY 3.0. Data by OpenStreetMap, under ODbL

We collected crow genetic information to compare with DNA from mosquito blood meals from local crow nestlings, breeders, and adult helpers. Nestlings (*n* = 72 nestlings from 30 nests in 2014) were banded, sampled, and monitored from all crow nests located along an established census route in Davis, California, during the 2014 breeding season [44, 45]. This study area encompassed the University of California, Davis campus, and the adjacent agricultural land (Fig. 1; described in [44]). Nestlings were sampled 1–36 days after hatching (mean ± SE = 20.8 ± 0.5 days), either within their nests or as young fledglings on branches immediately adjacent to nests. Nests were situated on lateral tree branches and accessed by boom lift. Nestlings that were first sampled < 18 days after hatching were resampled and banded > 22 days after hatching. Crows < 18 days old were too small for bands and were individually marked with a unique toenail clip; crows > 18 days old were marked with both a numbered USGS band and a unique color band [46]. Nestling age (accuracy: ± 3 days) was estimated based on an approximate hatch date (inferred from the shifting and probing behavior of incubating females, as well as size and feather development of nestlings) following criteria used in Townsend et al. [46]. Blood was collected (~ 150 μl) from the brachial or jugular vein of live nestlings or tissue samples from dead nestlings found in or under nests. Samples were preserved in Queen’s lysis buffer [47] until extraction for genetic analysis. Nestlings were returned to their nests immediately after sampling and were monitored for fledging 3–7 days per week along established census routes [26, 44, 45]. The coordinates for each nest, its activity period, and fate are available in Additional file 1.

Adult crows (breeders and helpers) underwent an annual molt (shedding and regrowth of feathers) on their breeding territories during the fledgling provisioning period from June through August each year. We were able, therefore, to extract DNA samples from passively molted feathers of 389 local adult crows to compare with our mosquito blood meal samples. We collected all feathers encountered in our focal territories along the established banding and census routes (Fig. 1) 3–7 days per week during the 2014 nestling and fledgling provisioning period (June through August 2014). We recorded the territory from which each feather was collected. A previous analysis of parentage and relatedness in this population showed that this sample of adult crow feathers included many parents of the nestlings sampled between 2012 and 2014 as well as non-breeding group members [48].

### Mosquito collection

Sixty artificial resting sites were deployed on 5 May 2014 in varying proximity to known crow nesting sites (Fig. 1). Resting sites were 5-gallon red plastic buckets, deployed horizontally within and along vegetation. Thirty-eight buckets were placed within known crow-breeding territories; 22 were placed in areas > 100 m from a known crow territory (see Additional file 2 for coordinates). When nest sites were known, the buckets were placed in the closest appropriate mosquito resting site (e.g. under low shrubs). Distance between the buckets and the nearest known crow nests ranged from 1 to 860 (mean ± SD = 147.7 ± 244.1) m. Individual nests varied in the timing of activity across the season. Nests were no longer considered active after nest failure or the nestlings fledged. Buckets were checked three mornings per week from 7 May 2014 to 3 September 2014, which included the majority of the crow nesting period and > 2 months after crows had completed their nesting cycle, depending on nesting initiation dates. Upon approaching each bucket, an insect net (Bioquip, Rancho Dominguez, CA, USA) was placed over the opening, and the bucket was turned upright to allow the insects to fly into the net. Captured mosquitoes were transferred into vials by handheld aspirator (Bioquip, Rancho Dominguez, CA, USA) and bucket number recorded. Mosquitoes were killed by cold in a – 80 °C freezer. A stereoscopic microscope was used to identify species and to determine whether they had recently taken a blood meal. Females with a visible blood meal were retained for blood meal host identification.

### Blood meal identification

DNA was extracted from individual mosquitoes using DNeasy Blood and Tissue kits (Qiagen, Valencia, CA, USA). To improve lysis, the blood meal contained within each mosquito was released by compressing the abdomen into the side of a microcentrifuge tube containing 20 µl of proteinase K using a clean pestle. Then, 200 ul of phosphate-buffered saline was added, and the sample was incubated overnight in a rocking incubator at 56 °C and 66 rpm, after which the extraction was continued according to the manufacturer’s protocol.

To identify blood meal hosts, the nested PCR approach described by Thiemann et al. [49] was used. In brief, the first PCR amplified a ~ 1900 base pair (bp) region of the tRNA-coding region flanking the mitochondrial gene cytochrome oxidase I (COI) using two sets of primers aligned to either avian or mammalian COI sequences. Then, using three sets of previously published primers designed to amplify a wide range of vertebrates [50, 51], the 658-bp barcoding region of the COI mitochondrial gene was amplified. PCR products were checked by gel electrophoresis for bands of the correct size (658 bp).

Samples containing the 658-bp target of interest were treated with ExoSAP-IT (Affymetrix Inc., Santa Clara, CA, USA) to eliminate unincorporated primers and dNTPs in preparation for Sanger sequencing. Sequencing was performed by the University of California, Davis College of Biological Sciences DNA Sequencing Facility (UC DNA). The Barcode of Life Data systems (BOLD) Identification Engine (http://boldsystems.org; [52]) was used to identify sequences. Samples yielded: (i) clean sequences identified to species by BOLD, (ii) unidentified mixed sequences where identified nucleotides had double peaks that resulted when mosquitoes fed on more than one host species and (iii) unamplified sequences. Only clean sequences as specified in (i) above were used in subsequent analyses.

### Molecular sexing and microsatellite analysis

To match mosquito blood meals to specific crow adults, helpers, and nestlings, DNA was extracted from nestling blood samples and adult feather tips using DNeasy tissue kits. All DNA samples from crow nestlings, adults, and mosquito blood meals of crow origin were assessed at diagnostic sex-linked alleles, using the P2/P8 sexing test primer set [53]. Samples were genotyped using a panel of 27 microsatellite loci developed for American crows [54, 55] and other corvids [56–58]. PCR conditions were described previously [48]. Individuals were scored at a minimum of 25 loci; 91% were scored at all loci. Mean allelic diversity was 10.1 ± 1.2 alleles/locus (range: 2–29 alleles/locus). No loci deviated significantly from Hardy-Weinberg equilibrium, and mean null allele frequency was 0.003 ± 0.003 (range: − 0.029 to 0.025). Locus characteristics including alleles/locus, observed and expected heterozygosity, and null allele frequencies are provided in Additional file 3.

Crow genotypes were matched between mosquito blood meals and specific individual crows using the “Identity” analysis in CERVUS 3.0.7 [59], allowing a maximum of four mismatches to account for genotyping errors or allele dropout. The “Identity” analysis was also used to identify and remove duplicate genotypes that occurred when more than one feather was collected and genotyped from specific local adults.

Results from a previous parentage analysis that utilized the maximum likelihood method in CERVUS (described in [48]) was used to evaluate the relationship between nestlings sampled in 2012–2014 and the adults that were identified as crow blood meals. In brief, all sampled adult males were specified as “potential fathers” and females as “potential mothers.” The potential typing error was specified at 5%, the proportion of sampled candidate parents at 65% and relatedness among 10% of candidate parents at 0.25. Candidate parents identified by CERVUS were accepted when confidence was high (> 95%) and the number of mismatches low (0–2). The combined exclusion probability of the first parent was very high (> 0.999998).

### Statistical analysis

Generalized linear mixed models (binomial distribution) were used to determine how date and proximity to active crow nests affected the likelihood that blood meals were of crow origin. Each mosquito blood meal with an identified avian host was the response, coded as 1 (crow) or 0 (non-crow). A total of five models were specified; model fit was compared using Akaike’s information criterion (AIC). First, three models were fit that included date of capture and the number of active crow nests (e.g. from the start of the incubation period until fledging or failing) on that date within 10, 50 or 100 m of the site of mosquito collection. Models were fit with varying distance thresholds because of uncertainty about flight distances traversed by blood-engorged female *Culex* seeking a resting site. A fourth model was fit that included date alone as a predictor, and a final null model included no predictor variables. All five models included a random effect for trap location to account for the non-independence of mosquitoes captured at the same resting bucket and the fact that some buckets contributed disproportionately to the total number of captures. Models were fit using the ‘glmer’ function in the ‘lme4’ package [60]. All AICc values were compared using the ‘MuMIn’ package [61] in Program R version 4.0.2 [62].

## Results

### Blood meal species identification

**Fig. 2:**
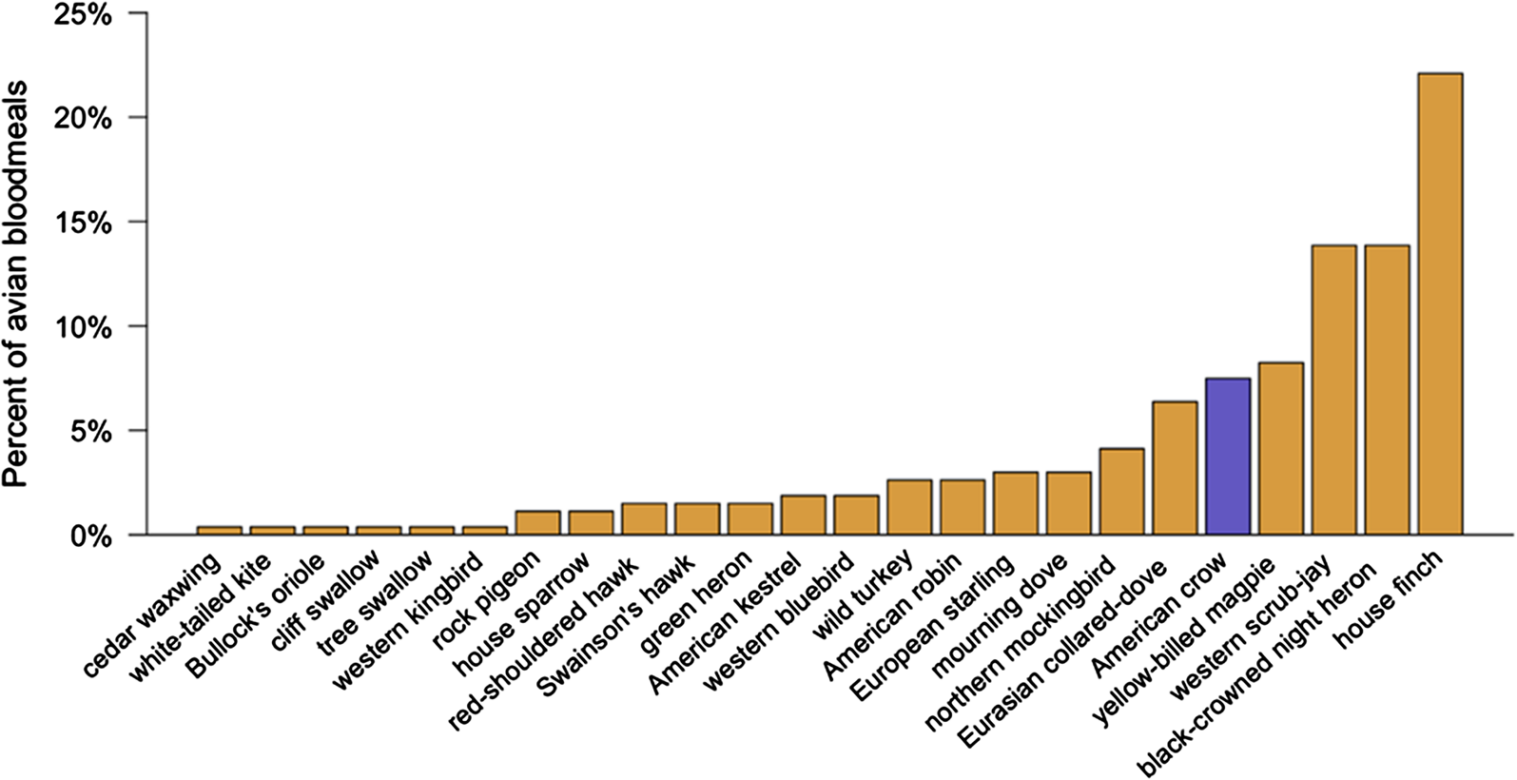
Percentage of *Cx. pipiens* and *Cx. tarsalis* blood meals originating from each avian host species (*n* = 267). American crows (highlighted in blue) were hosts for 6.7% of the total number (*n* = 297) of bloodfed *Culex.* Other species included cedar waxwing *Bombycilla cedrorum*; white-tailed kite *Elanus leucurus*; Bullock’s oriole *Icterus bullockii*; cliff swallow *Petrochelidon pyrrhonota*; tree swallow *Tachycineta bicolor*; western kingbird *Tyrannus verticalis*; rock pigeon *Columba livia*; house sparrow *Passer domesticus*; red-shouldered hawk *Buteo lineatus*; Swainson’s hawk *Buteo swainsoni*; green heron *Butorides virescens*; American kestrel *Falco sparverius*; western bluebird *Sialia mexicana*; wild turkey *Meleagris gallopavo*; European starling *Sturnus vulgaris*; northern mockingbird *Mimus polyglottos*; Eurasian collared-dove *Streptopelia decaocto*; yellow-billed magpies *Pica nuttalli*; California scrub-jay *Aphelocoma californica*; black-crowned night-heron *Nycticorax nycticorax*

Overall, 569 bloodfed female mosquitoes comprising seven species were collected. Sequencing results identified blood meal hosts from 398 (Additional file 4) individual mosquitoes. Of the unidentified blood meals, 25 had mixed sequences (4 from *Cx. tarsalis* and 1 *Cx. pipiens*), indicating multiple blood meal hosts, and 146 failed to amplify. Among the identified blood meals, 297 were either from *Cx. tarsalis* or *Cx. pipiens*, and there was a single collection of *Cx. stigmatosoma* Dyar, ornithophagic species most likely to feed on crows. The percentages of *Culex* blood meals originating from each avian host species are shown in Fig. 2. The other mosquito species collected were *Aedes sierrensis* Ludlow, *Anopheles franciscanus* McCraken, *Anopheles freeborni* Aitken and *Culiseta incidens* Thomson. Overall 96% (*n* = 101) of the blood meals from these species were mammalian in origin, and none were from crows.

Twenty blood meals were attributable to crows, which constituted 6.7% of the total number of blood meals identified from *Cx. tarsalis* and *Cx. pipiens*. All of the crow blood meals occurred between 1 May and 18 June, before nestlings had fledged from their nests. The mean percentage of blood meals of crow origin per week during the nesting period was 19.1% ± 0.7 (SE) and 0% thereafter. Percentage of blood meals of crow origin declined significantly across weeks [glm (binomial distribution) with proportion of crow blood meals per week as the response, weighted by sample size of blood meals analyzed each week; β ± SE = − 0.34 ± 0.09; *p* < 0.001)].

**Fig. 3:**
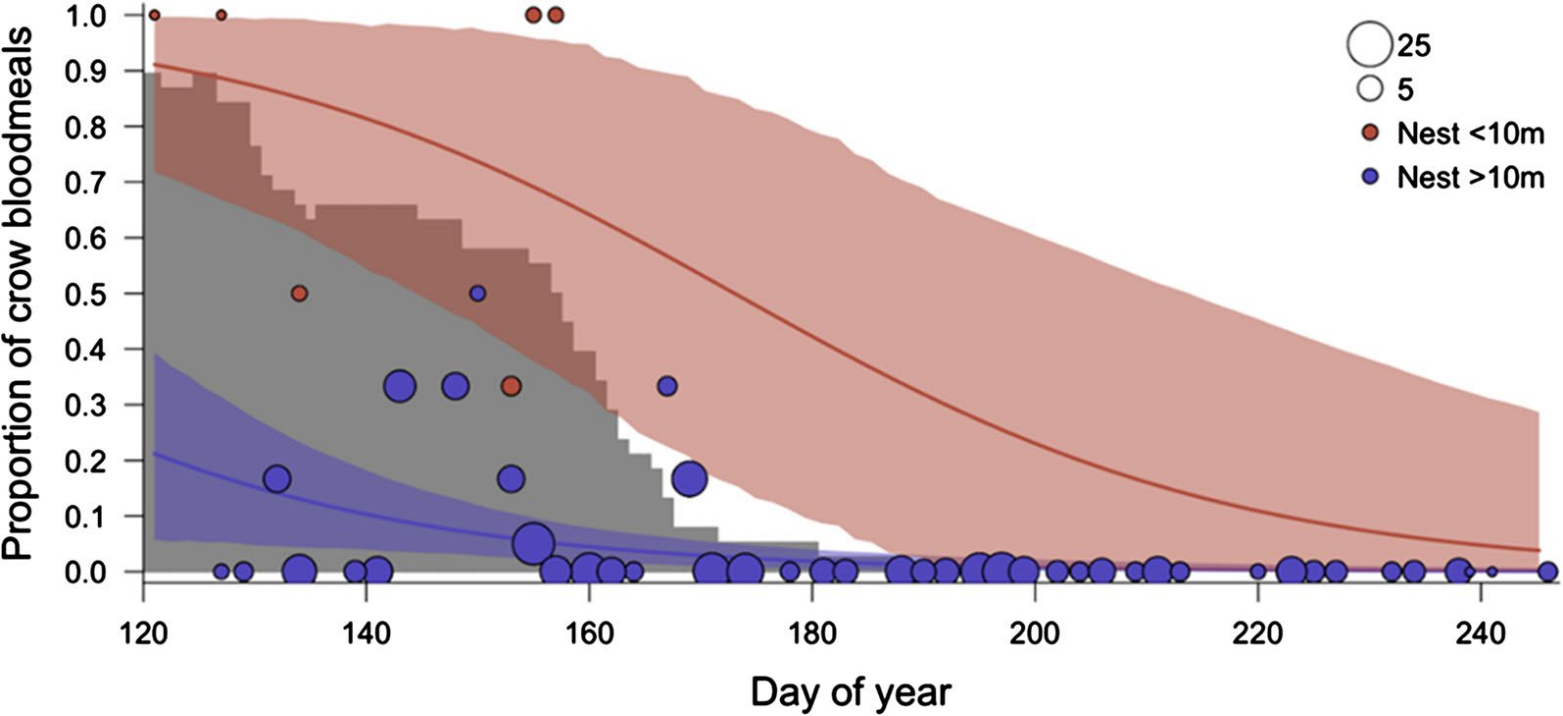
The proportion of *Culex* blood meals of crow origin declined from 5 May (day 125) to 3 September (day 246) 2014 and with distance from active crow nests (distances > 10 m). The gray histogram indicates the number of active crow nests on each day of the season. The size of the circles depicts the number of bloodfed *Culex* collected on a single day, grouped by proximity to an active crow nest (i.e. >/< 10 m from a crow nest). No blood meals were of crow origin after 18 June 2014 (day 169), after which all focal nestlings had fledged from nests

Both date and distance to an active crow nest affected the likelihood of a crow blood meal (Table 1). There was strong model support for the effect of active crow nests within 10 m or 50 m of the point of mosquito capture on the likelihood of a crow blood meal (cumulative support for these two models = 0.98), although support was considerably stronger for the 10 m cutoff than the 50 m cutoff (**Δ**AIC = 2.59). There was no evidence for an effect of nests when the distance was extended to a 100 m radius on the likelihood of a crow blood meal (**Δ**AIC = 8.83). The model fit details for the 10 m and 50 m cut-offs are shown in Table 2. The odds that crows were hosts of a *Culex* blood meal were 38.07 times greater within 10 m of an active nest (*p* = 0.001) and 12.08 times greater within 50 m of an active nest (*p* = 0.002) than when nests were farther away. In both models, the likelihood of a crow blood meal declined significantly with date (*p* < 0.05). The proportion of blood meals of crow origin as a function of date and distance from active crow nests (based on the 10 m model) is shown in Fig. 3. The fit lines are maximum likelihood estimates. The confidence intervals for the two levels (yes/no for nest < 10 m) were determined by pulling 500,000 samples from the posterior distribution of the fit model using the ‘mvrnorm’ function from the ‘MASS’ package [63] and then determining the 90% highest posterior density interval using the ‘rethinking’ package [64] in R.

**Table 1:** Crow blood meal likelihood model comparison

Model	*K*	ΔAIC_c_	*W*_i_	Log likelihood
~ Active nest < 10 m + day of year	4	0.00	0.77	− 49.46
~ Active nest < 50 m + day of year	4	2.59	0.21	− 50.76
~ Active nest < 100 m + day of year	4	8.83	0.01	− 53.88
~ Day of year	3	9.29	0.01	− 55.14
~ Intercept only	2	28.13	0.00	− 65.57

Likelihood estimates of crow blood meals as a function of date and proximity to active crow nests. Each model included a random effect for bucket identity

*K* number of parameters, *ΔAIC*_*c*_ delta AIC_c_, *w*_*i*_ cumulative model weight, *LL* log likelihood

**Table 2:** Output from generalized linear mixed models

Predictors	Likelihood of crow DNA (10 m)			Likelihood of crow DNA (50 m)		
	Odds ratios	CI	*P*	Odds ratios	CI	*P*
Intercept	0.02	0.01–0.07	< 0.001	0.02	0.00–0.06	< 0.001
Day of year (standardized)	0.26	0.11–0.64	0.003	0.28	0.11–0.70	0.007
Active nest < 10 m	38.07	4.43–327.47	0.001			
Active nest < 50 m				12.08	2.42–60.16	0.002
Observations	297			297		
Marginal R2/conditional R2	0.390/0.516			0.352/0.529		

Generalized linear mixed models tested the likelihood that blood meals were of crow origin as a function of date and proximity to active crow nests (within 10 m or 50 m of active nests). The marginal and conditional R2 give approximations of R2 (modified for mixed models) for the fixed effects (marginal) and the full model including random effects (conditional)

**Fig. 4:**
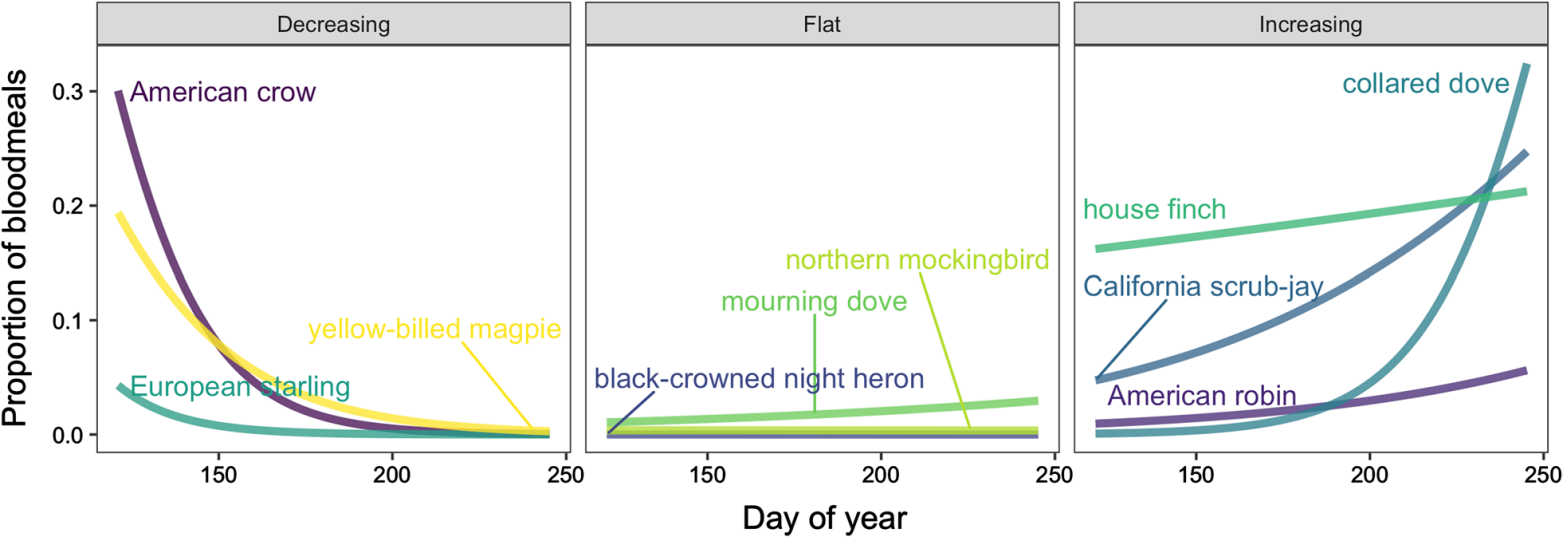
The proportion of *Culex* blood meals as a function of date varied among avian host species

The relationship between date and origin of *Culex* blood meals varied among avian host species. The proportions of blood meals originating from some of the most common host species are shown in Fig. 4. For some species, particularly American crows and yellow-billed magpies (*Pica nuttalli*), proportional representation in *Culex* blood meals declined between May and September; for others (notably California scrub-jays and collared doves), the reverse was true. The lines shown are maximum likelihood estimates from separate models fit for each species. As above, these host-specific models were fit using generalized linear mixed models (binomial distribution), with each mosquito blood meal with an identified avian host [coded as 1 (specific avian host species) or 0 (other host)] as the response, date as a fixed effect and trap location as a random effect.

### Crow sampling and individual identity analysis

Overall, 75 active crow nests were identified in our study location in 2014. Nestlings were sampled from 30 of these nests, 20 of which fledged offspring and 10 that failed in the nestling stage. Among the remaining 45 nests, 36 failed in the egg or early nestling stage before the chicks were genetically sampled. The remaining nine nests along the survey routes were too high to reach by boom lift and were not included. In total, DNA was collected from crow offspring originating from 40% of the broods produced by local crow family groups in 2014. In addition, feather DNA from 389 unmarked individual adults (after excluding duplicate genotypes) was collected from the study area during the fledgling provisioning period from June through August 2014.

Overall, 17 of the 20 mosquito blood meals of crow origin had matched genotypes (Additional file 5). Ten of these 17 samples were matched by the “Identity” function in CERVUS to the genotypes of specific crows. Seven genotypes matched those of local crow nestlings, all from nests < 50 m from the mosquito collection point. Three samples belonged to local adults, two of which were identified by parentage analysis in CERVUS as putative mothers of local broods (also with nests < 50 m from the mosquito collection point). The third sample from an adult female was not identified as a parent of any of the broods sampled; this bird therefore could have either been a breeder of an unsampled brood or a non-breeding helper at the nest. In total, therefore, seven of ten samples of known identity belonged to local nestlings, two of ten samples belonged to local putative female breeders, and one of ten belonged to an adult female non-breeder or breeder with an unsampled brood. The seven genotypes that were not matched could have originated from unsampled nestlings (that were not sampled or whose nests failed prior to acquisition of genetic samples), adult parents of unsampled nestlings or adult non-breeders (helpers). For five of the seven unidentified individuals, at least one local nest (< 50 m of the mosquito collection site) failed prior to genetic sampling. Six of these seven unidentified individuals were female by diagnostic sex-linked alleles; the seventh could not be reliably sexed.

## Discussion

Despite the high frequency with which they succumb to WNV and their high viral titers after infection, the degree to which crows serve as mosquito hosts and, therefore, their contribution to viral amplification remains controversial [5, 12, 17]. Our data indicate that crows may be an important host for *Culex* mosquitoes in the early breeding season, particularly when females are brooding nestlings. Several lines of evidence support this hypothesis. The mean percentage of blood meals of crow origin was 19% in the nesting period (1 May–18 June 2014), but 0% in the weeks after fledging (19 June–1 September 2014). Moreover, the likelihood of a crow blood meal increased with proximity to an active nest: the odds that crows hosted a *Culex* blood meal were 38.07 times greater within 10 m of an active nest than > 10 m from a nest. Overall, 90% of crow blood meals that could be matched to a genotype of a specific crow belonged to either nestlings in these nests or their mothers, which would be incubating or brooding the nestlings during this time period. Six of the seven genotypes that could not be attributed to sampled birds belonged to females, a sex bias likely due to mosquitoes targeting incubating or brooding females [24]. In concert, these data suggest that breeding crows do serve as hosts for *Culex* in the initial stages of the WNV spring enzootic cycle. Given their high viremia, infected crows could thereby contribute to the re-initiation and early amplification of the virus, increasing its availability as mosquitoes shift to other, moderately competent, later-breeding avian hosts.

For a host to be important to the WNV cycle, it must produce a viral titer high enough to infect a mosquito vector, survive long enough to transmit the infection and have sufficient contact with vector species [23]. Although crows mount among the highest viremias reported for any host and rank highly on the vertebrate host-competence index [6, 65], the reported infrequency with which they are fed upon by mosquitoes would suggest that they are less important for transmission than less competent, but more frequently fed upon hosts [12]. Nevertheless, crows clearly have substantial exposure to the virus. In the years of our study, for example, WNV was the most prevalent disease detected in the Davis, California, crow population, with 35.8% of dead crows testing positive for WNV [26]. Some of these WNV-positive birds were nestlings [66], with carcasses recovered within or immediately under the nest (8/24 WNV-positive carcasses; unpublished data). On a national scale, regional crow population reductions of > 45% were observed after initial WNV emergence [9, 67], and (unlike most other passerine species) crow populations have not recovered to pre-WNV numbers [68]. Such changes in crow abundance have been used to model spatio-temporal changes in WNV prevalence [69]. These patterns beg the question of why the representation of crows in mosquito blood meals is so low in most studies.

The tight link between nest proximity and likelihood of being a mosquito host indicates that mosquito collection site locations probably have contributed to the scarcity of crow hosts detected in other studies. We found that 19% of the blood meals collected during the crow breeding season originated from crows, but the effect was highly localized in space: when date was held at May 30th, a mosquito captured in a bucket with no active crow nests within 10 m had only a 7.3% chance of having fed on a crow (95% CI = 2.9–13.7%). In contrast, when a crow nest was active within 10 m of the capture site, the same mosquito had a 58.9% chance of having fed on a crow (95% CI = 40.0–77.3%). The pattern was similar, although not as strong, for the 50 m model: a mosquito had only a 5.9% chance of having fed on a crow (95% CI = 1.7–12.8%) with no active crow nests within 50 m, but a 36.3% chance of a crow blood meal when a crow nest was active within 50 m of the capture site (95% CI = 17.4–59.8%).

This study specifically targeted crow territories for mosquito collection sites; more than half of the collection buckets (63%) were placed within known crow breeding territories, and the remainder was placed > 100 m distant from known crow territories (Fig. 1). These crow territories were distributed regularly throughout the residential and agricultural landscape of Davis, California, with nests generally occurring in tall coniferous trees adjacent to lawns or other grassy areas. All crow territories encompassed human habitation, agricultural fields and/or grazing land; no crow territories were established within large patches of contiguous forest or scrubland. Therefore, crows might be underrepresented in other studies if mosquito collection is focused on microhabitats underutilized by nesting crows (e.g. forested or otherwise undeveloped/natural areas). Indeed, in two previous studies in the same study population (neither of which were focused on crow nesting habitat), none of the mosquito blood meals collected between 2007 and 2009 were from crow hosts [37, 70]. In contrast, bloodfed *Culex* collected in sites where nesting crows were observed did feed on crow hosts in a Sutter County, California, population during the crow breeding season (May–June): 17.2% and 8.2% of *Cx. pipiens* and *Cx. tarsalis* blood meals, respectively, were of crow origin in this study [22].

Proportional abundance of avian hosts is likely to play an important role in their representation within mosquito blood meals and therefore the efficiency of WNV transmission. Even when mosquito sampling sites are established within crow territories, mosquitoes might be more likely to encounter and feed upon species with a greater relative abundance, smaller territory size, greater local nest density and nest structure and position to intercept host-seeking females. We were unable to test this hypothesis as we did not estimate the relative abundance, timing of breeding or nest locations of other breeding species in our study area. The contribution of crows to *Culex* blood meals—and therefore to WNV transmission—is likely to be lower in areas where their relative abundance is lower.

Seasonal changes in proportional representation in *Culex* blood meals varied among avian hosts (Fig. 4). Representation of crows declined between May and September, in a pattern matching its breeding period (Fig. 3), whereas other species increased in representation (e.g. California scrub-jays and collared doves) and others remained stable throughout the season (e.g. house finches). Crows are among the earliest breeders in this area, initiating nest-building in late March and early April. Variation in patterns of host representation could reflect their timing of peak reproduction throughout the summer, if mosquitoes target vulnerable, nest-bound hosts. Although data on the timing of breeding of other avian hosts within this population are needed to evaluate this hypothesis, similar temporal host shifts corresponding with timing of breeding have been reported among avian hosts in other areas [24, 25, 37].

Although we only detected crows in *Culex* blood meals during their nesting season, they clearly serve as hosts later in the season as well, as indicated by widespread crow representation in WNV surveillance dead bird programs in the post-breeding months (August–October; [1, 3, 4, 29]). These transmission events are unlikely to be driven by bird-to-bird transmission, of which there is no evidence in this population [27]. Sampling design and changes in crow behavior could account for the absence of crows in late-season blood meal samples. In the nesting season, crows are distributed regularly in their territories throughout appropriate breeding habitat, which we targeted in our sampling design. Vulnerable, nest-bound crows would be widely available across these habitats to host-seeking mosquitoes during this period. After the breeding season and within the peak WNV transmission season (e.g. August–October), however, resident crows in this population congregate at communal roosts of 100–400 resident individuals, often in more urban settings [71]. Crows at these roosts might be important hosts for crepuscular *Culex* mosquitoes, which generally seek hosts at night. However, these roosts are discrete, unpredictable and continually shifting in location, and none were sampled specifically in our study. Therefore, the absence of crows from late-season samples could be due, at least in part, to the absence of roost locations in our mosquito collection sites.

## Conclusions

In temperate climates where cold winters suppress mosquito activity, the WNV transmission cycle needs to be re-initiated each spring [24]. Where nesting crows serve as mosquito hosts in spring (as they did in the Davis, California, population), this highly competent host could contribute to local amplification of WNV, which is then circulated when mosquitoes shift to later-breeding, less competent avian host species. However, given that the utilization of crows as hosts was highly localized around active nests, it is unclear how widely their contribution to WNV transmission might extend across the landscape. A similar localized effect was indicated by another Davis, California, study, which showed that the odds of detecting infected mosquitoes were higher at residences where dead crows were reported [11]. Moreover, the extent of the contribution of crows as hosts is likely to vary with relative abundance of other hosts and the timing of their nesting cycles, which will vary among populations. Roosting behavior could also play an important role in crow WNV transmission events, and the underrepresentation of roosts in mosquito sampling designs could contribute to the underrepresentation of crows among mosquito blood meal studies [18, 70, 72, 73]. Future work that examines the contribution of crows to mosquito blood meals as a function of the timing of breeding of other hosts and proximity to communal roosts would better elucidate the factors mediating the contribution of this highly competent host to the WNV cycle.

## Supplementary Information

**Additional file 1**: American crow nest locations, activity period and fate. Latitude and longitude of each nest location is provided, additional columns provided information on whether the chicks were sampled, the ultimate fate of the nest, and the activity dates.

**Additional file 2**: Resting mosquito collection bucket locations. Latitude and longitude of all buckets placed in the field.

**Additional file 3**: Characteristics of microsatellite loci use for this study. Data table containing locus characteristics including alleles/locus, observed and expected heterozygosity, and null allele frequencies.

**Additional file 4**: Complete list of identified blood meal hosts. Data table containing mosquito species, collection date and location, and identified blood meal hosts for all identified blood meals.

**Additional file 5**: Identified crow and mosquito genotypes. Data table containing a list of all crow genotypes collected from: crow blood samples, collected feathers, and mosquito blood meals.

## Data Availability

All data generated or analyzed during this study are included in this published article [and its Additional files].
