# Supplementary material for: Mosquito blood-feeding patterns and nesting behavior of American crows, an amplifying host of West Nile virus
Source: Parasit Vectors. 2021 Jun 22;14:331. doi: 10.1186/s13071-021-04827-x (PMC8220764; doi:10.1186/s13071-021-04827-x)
Supplement: Supplementary file 3 — Additional file 3: Characteristics of microsatellite loci use for this study. Data table containing locus characteristics including alleles/locus, observed and expected heterozygosity, and null allele frequencies. [file 13071_2021_4827_MOESM3_ESM.docx]

Additional File 3. Characteristics of microsatellite loci used for this study

| **Locus name** | **Reference** | **K** | **HObs** | **HExp** | **HW** | **F(Null)** |
| --- | --- | --- | --- | --- | --- | --- |
| ApCo18 | [[1]](https://paperpile.com/c/ZLP2KW/tdawA) | 8 | 0.757 | 0.757 | NS | -0.0002 |
| ApCo30 | [[1]](https://paperpile.com/c/ZLP2KW/tdawA) | 8 | 0.556 | 0.581 | NS | 0.024 |
| ApCo31 | [[1]](https://paperpile.com/c/ZLP2KW/tdawA) | 16 | 0.807 | 0.811 | NS | 0.003 |
| ApCo40 | [[1]](https://paperpile.com/c/ZLP2KW/tdawA) | 5 | 0.198 | 0.197 | NS | -0.0109 |
| ApCo95 | [[1]](https://paperpile.com/c/ZLP2KW/tdawA) | 8 | 0.766 | 0.752 | NS | -0.0097 |
| Cb04 | [[2]](https://paperpile.com/c/ZLP2KW/Cd4qQ) | 6 | 0.752 | 0.775 | NS | 0.0171 |
| Cb05 | [[2]](https://paperpile.com/c/ZLP2KW/Cd4qQ) | 28 | 0.753 | 0.788 | NS | 0.0243 |
| Cb06 | [[2]](https://paperpile.com/c/ZLP2KW/Cd4qQ) | 8 | 0.734 | 0.743 | NS | 0.0048 |
| Cb07 | [[2]](https://paperpile.com/c/ZLP2KW/Cd4qQ) | 4 | 0.319 | 0.317 | NS | 0.004 |
| Cb08 | [[2]](https://paperpile.com/c/ZLP2KW/Cd4qQ) | 7 | 0.633 | 0.638 | NS | 0.0015 |
| Cb10 | [[2]](https://paperpile.com/c/ZLP2KW/Cd4qQ) | 7 | 0.762 | 0.723 | NS | -0.0286 |
| Cb13 | [[2]](https://paperpile.com/c/ZLP2KW/Cd4qQ) | 9 | 0.831 | 0.854 | NS | 0.0132 |
| Cb14 | [[2]](https://paperpile.com/c/ZLP2KW/Cd4qQ) | 9 | 0.719 | 0.739 | NS | 0.0121 |
| Cb16 | [[2]](https://paperpile.com/c/ZLP2KW/Cd4qQ) | 5 | 0.497 | 0.479 | NS | -0.0247 |
| Cb17 | [[2]](https://paperpile.com/c/ZLP2KW/Cd4qQ) | 10 | 0.694 | 0.705 | NS | 0.0106 |
| Cb20 | [[2]](https://paperpile.com/c/ZLP2KW/Cd4qQ) | 6 | 0.133 | 0.14 | NS | 0.0253 |
| Cb22 | [[2]](https://paperpile.com/c/ZLP2KW/Cd4qQ) | 8 | 0.731 | 0.739 | NS | 0.0034 |
| Ck1B5D | [[3]](https://paperpile.com/c/ZLP2KW/0gwRv) | 11 | 0.708 | 0.719 | NS | 0.005 |
| Ck1B6G | [[3]](https://paperpile.com/c/ZLP2KW/0gwRv) | 14 | 0.836 | 0.848 | NS | 0.0079 |
| CoBr02 | [[4]](https://paperpile.com/c/ZLP2KW/CtS9k) | 10 | 0.697 | 0.692 | NS | -0.0073 |
| CoBr08 | [[4]](https://paperpile.com/c/ZLP2KW/CtS9k) | 8 | 0.667 | 0.66 | NS | -0.0049 |
| CoBr12 | [[4]](https://paperpile.com/c/ZLP2KW/CtS9k) | 11 | 0.777 | 0.781 | NS | 0.0017 |
| CoBr19 | [[4]](https://paperpile.com/c/ZLP2KW/CtS9k) | 20 | 0.926 | 0.917 | NS | -0.0059 |
| CoBr22 | [[4]](https://paperpile.com/c/ZLP2KW/CtS9k) | 11 | 0.678 | 0.697 | NS | 0.0144 |
| CoBr25 | [[4]](https://paperpile.com/c/ZLP2KW/CtS9k) | 5 | 0.29 | 0.288 | NS | -0.0004 |
| CoBr36 | [[4]](https://paperpile.com/c/ZLP2KW/CtS9k) | 29 | 0.844 | 0.854 | NS | 0.005 |
| PnuC222w | [[5]](https://paperpile.com/c/ZLP2KW/8Lq2Z) | 2 | 0.204 | 0.204 | NS | 0.0004 |

CK = number of alleles; HObs = observed heterozygosity; HExp = expected heterozygosity; HW = significance (after Bonferroni correction) of deviation from Hardy-Weinberg equilibrium; F(Null)= estimated null allele frequency. NS = not significant. Summary statistics estimated in CERVUS 3.0.

**References**

[1. Stenzler LM, Fitzpatrick JW. Isolation of microsatellite loci in the Florida scrub-say *Aphelocoma coerulescens*. Mol Ecol Notes. 2002;2:547–50.](http://paperpile.com/b/ZLP2KW/tdawA)

[2. Verdugo C, Clark AM, Prakoso D, Kramer LD, Long MT. Multiplexed microsatellite loci in American crow (*Corvus brachyrhynchos*): a severely affected natural host of West Nile virus. Infect Genet Evol. 2012;12:1968–74.](http://paperpile.com/b/ZLP2KW/Cd4qQ)

[3. Tarr CL, Fleischer RC. Primers for polymorphic GT microsatellites isolated from the Mariana crow, *Corvus kubaryi*. Mol Ecol. 1998;7:253–5.](http://paperpile.com/b/ZLP2KW/0gwRv)

[4. Schoenle LA, Townsend AK, Lovette IJ. Isolation and characterization of microsatellite loci in a cooperatively breeding corvid, the American crow (*Corvus brachyrhynchos*). Mol Ecol Notes. 2007;7:46–8.](http://paperpile.com/b/ZLP2KW/CtS9k)

[5. Ernest HB, Well JA, Kurushima JD. Development of 10 microsatellite loci for yellow-billed magpies  *Pica nuttalli*  and corvid ecology and West Nile virus studies. Mol Ecol Resour. 2008;8:196–8.](http://paperpile.com/b/ZLP2KW/8Lq2Z)
